# Bovine Leukemia Virus Infection Affects Host Gene Expression Associated with DNA Mismatch Repair

**DOI:** 10.3390/pathogens9110909

**Published:** 2020-10-30

**Authors:** Lanlan Bai, Tomoya Hirose, Wlaa Assi, Satoshi Wada, Shin-nosuke Takeshima, Yoko Aida

**Affiliations:** 1 Photonics Control Technology Team, RIKEN Center for Advanced Photonics, Wako 351-0198, Japan; lanlan.bai@riken.jp (L.B.); wlaa.pharma92@yahoo.com (W.A.); swada@riken.jp (S.W.); takesima@jumonji-u.ac.jp (S.-n.T.); 2 Viral Infectious Diseases Unit, RIKEN, Wako 351-0198, Japan; kamaboko517tmp@gmail.com; 3 Laboratory of Viral Infectious Diseases, Department of Medical Genome Sciences, Graduate School of Frontier Science, The University of Tokyo, Tokyo 113-8657, Japan; 4 Department of Food and Nutrition, Faculty of Human Life, Jumonji University, Niiza 352-0017, Japan; 5 Nakamura Laboratory, Baton Zone Program, RIKEN Cluster for Science, Technology and Innovation Hub, Wako 351-0198, Japan

**Keywords:** DNA mismatch repair, bovine leukemia virus, proviral load, viral protein expression, gene expression, lymphoma stage

## Abstract

Bovine leukemia virus (BLV) causes enzootic bovine leukosis, a malignant form of B-cell lymphoma, and is closely related to human T-cell leukemia viruses. We investigated whether BLV infection affects host genes associated with DNA mismatch repair (MMR). Next-generation sequencing of blood samples from five calves experimentally infected with BLV revealed the highest expression levels of seven MMR genes (*EXO1*, *UNG*, *PCNA*, *MSH2*, *MSH3*, *MSH6*, and *PMS2*) at the point of peak proviral loads (PVLs). Furthermore, MMR gene expression was only upregulated in cattle with higher PVLs. In particular, the expression levels of *MSH2*, *MSH3*, and *UNG* positively correlated with PVL in vivo. The expression levels of all seven MMR genes in pig kidney-15 cells and the levels of *PMS2* and *EXO1* in HeLa cells also increased tendencies after transient transfection with a BLV infectious clone. Moreover, MMR gene expression levels were significantly higher in BLV-expressing cell lines compared with those in the respective parental cell lines. Expression levels of *MSH2* and *EXO1* in BLV-infected cattle with lymphoma were significantly lower and higher, respectively, compared with those in infected cattle in vivo. These results reveal that BLV infection affects MMR gene expression, offering new candidate markers for lymphoma diagnosis.

## 1. Introduction

Each cell typically sustains tens of thousands of DNA lesions daily as a result of exposure to ultraviolet radiation, carcinogens, or viruses, among other factors [1,2]. Despite this high frequency of damage, the integrity of the genetic system is maintained owing to the presence of molecular mechanisms that continuously monitor and repair DNA damage. DNA repair mechanisms can be broadly classified into five categories: mismatch repair (MMR) [3], base excision repair, nucleotide excision repair, homologous recombination repair, and nonhomologous end joining [2].

MMR is a mechanism that corrects mismatches caused by the incorporation of incorrect bases during DNA mutation or replication [4]. When a DNA mismatch occurs, the MutS complex (MSH2-MSH6) protein recognizes and binds to the mismatch site [4,5]. The MutL complex (PMS2/MLH1) protein then binds to the mismatch site in an ATP-dependent manner, as an entry point for exonuclease 1 (EXO1), thereby sliding along the DNA until reaching a position about 25 bases away from the binding site [6,7,8]. Proliferating cell nuclear antigen (PCNA) and replication factor C are then allowed to bind, and the DNA is nicked by endonuclease activity [8]. Furthermore, replication protein A binds to single-stranded DNA to enhance the stability [4], and the DNA is replicated by DNA polymerase δ assisted by PCNA [9]. The product is ultimately obtained, and the DNA is repaired by ligation [6]. Notably, MMR proteins are involved in cell cycle regulation, induction of apoptosis [4,10,11,12], and antibody diversification processes such as the class switch recombination of immunoglobulin (Ig) genes and somatic mutation [13,14,15]. Generally, somatic hypermutation and class switch recombination of Ig genes occur in germinal center B cells. The nature of these genetic remodeling events makes these cells uniquely vulnerable to malignant transformation [16].

Enzootic bovine leukosis (EBL), as a malignant form of B-cell lymphoma, occurs following bovine leukemia virus (BLV) infection, with a worldwide occurrence contributing to serious losses in the cattle industry [17]. Nearly 70% of BLV-infected cattle are asymptomatic carriers, with the remaining 30% of infections progressing to persistent lymphocytosis, characterized by polyclonal expression of the non-neoplastic CD5+ B lymphocyte population. Less than 5% of infected cattle develop B-cell leukemia/lymphoma after a long latency period. However, the mechanism conferring protection against EBL for most BLV-infected cattle remains unclear. 

BLV is a retrovirus [17,18] that is closely related to human T-cell leukemia virus type 1 (HTLV-1), which is the causative agent of adult T-cell leukemia [19]. Therefore, BLV-induced B cell lymphoma provides a useful model for obtaining insight into the biology of various human B cell lymphomas, including Epstein-Barr virus- and human herpesvirus-8-induced B cell lymphoma [20]. The BLV genome encodes essential genes (e.g., *gag*, *pro*, *pol*, and *env*) that produce infectious virions, as well as regulatory genes (*tax*, *rex*) and accessory genes (*R3*, *G4*) [17,18]. Tax protein is an important factor in elucidating the mechanism of leukemia pathogenesis as it is involved in the immortalization and proliferation of tax-expressing cells [17], and has a significant impact on diverse cellular functions such as transcription, signal transduction, cell growth, and stress and immune responses [21]. Retroviruses have been reported to efficiently repair integration site damage, maintain cellular homeostasis, and induce transcription of the viral genome [22,23,24,25,26]. In addition, host cells that evade apoptosis can sense DNA damage due to viral infections to elicit an immune response [24,26,27,28,29], and some viruses can induce the apoptosis of central cells responsible for immune functions, resulting in abnormalities of the DNA repair mechanisms to inhibit the host immune response and maintain the infection [30,31]. Retroviruses have been reported to be capable of infecting different cell types and induce different types of damage to these cells. However, the relationship between retroviruses and DNA repair mechanisms has not yet been clarified in detail.

The BLV proviral load (PVL), which represents the amount of retroviral genome integrated in the host genome, correlates strongly not only with the BLV infection capacity, as assessed by syncytium formation [32,33], but also with BLV disease progression [32,34,35]. Additionally, the BLV PVL is considered a useful index for estimating the transmission risk [36]. One study detected BLV provirus in milk samples of infected cows only when the PVL in the blood samples exceeded 10,000 copies/10^5^ cells [37]. Cows with a PVL higher than 14,000 copies/10^5^ cells in their blood were found to secrete BLV in the nasal mucus [38]. These findings suggest that a PVL of approximately 10,000 copies/10^5^ cells is relatively high and indicates the spread of BLV throughout the body. We previously reported that the BLV PVL is strongly associated with bovine leukocyte antigen (*BoLA*)-*DRB3* alleles [35,39,40]. In addition, the PVL was reported to be correlated with the progression of EBL pathology, and a high PVL in cattle increased the risk of spreading the infection [34,41]. However, several BLV-infected cattle do not show such a correlation between *BoLA-DRB3* alleles and the PVL. This suggests that a novel host factor in addition to the *BoLA* class II gene regulates the PVL in the body upon infection.

To identify these regulating factors, in the present study, we established an experimental infection cattle model of BLV and performed next-generation sequencing (NGS) of blood samples to identify host genes with altered expression according to an increase or decrease in the PVL. As the relationship between MMR genes and retroviruses has not yet been clarified, our aim was to clarify whether BLV infection impacts MMR gene regulation.

## 2. Results

### 2.1. Comprehensive Gene Analysis of Cattle Experimentally Infected with BLV in Vivo

Many risk factors associated with the PVL are known to increase the infection rate of BLV [34,35,37,38,39]. The *BoLA-DRB3* allele has been associated with individual differences among cattle in their response to BLV infection. Therefore, we used calves of the same age who were all confirmed to harbor the same *BoLA-DRB3* allele for the NGS analysis to avoid the impact of individual differences. In addition, to minimize the effects of physical conditions, the calves were housed in the same p2-level barn with controlled temperature and humidity and were fed the same fodder. 

**Figure 1:**
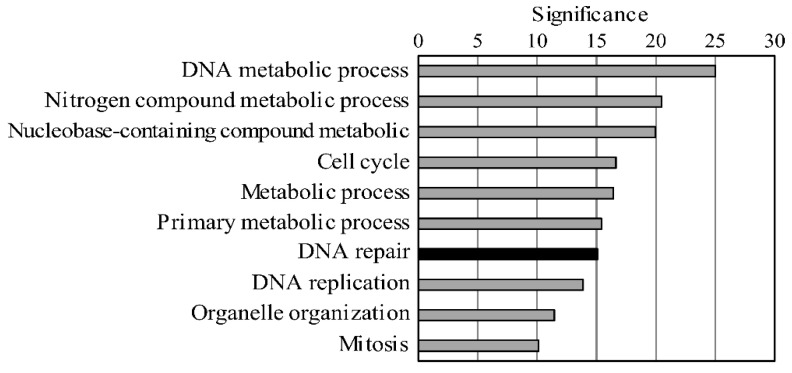
The top ten up regulated biological functions due to BLV infection. Gene Ontology (GO) analysis was performed using the gene expression analysis free site PANTHER in the list of 906 genes that had up regulated due to BLV infection. Among the biological functions obtained by the analysis, the top ten functions with the highest significance are shown.

To analyze changes in gene transcript expression only due to BLV infection, we experimentally infected five BLV-negative calves carrying the susceptible alleles BoLA-DRB3*1601/1601, which have been related to a high PVL in BLV infection. RNA was extracted from the blood of the five calves both before and after experimental BLV infection; thus, the pre-infection samples served as the negative control for each cattle. The host genes affected by BLV infection were then identified by comparison of the NGS data from the same calves before and after BLV infection. In total, 906 and 1328 genes were found to be upregulated and downregulated following infection, respectively, with the peak PVL observed in the third week after BLV infection. The top 10 genes with upregulated expression after infection were further selected for functional analysis according to Gene Ontology (GO) enrichment (Table 1 and Figure 1). Notably, colon cancer cell lines deficient in MMR have been shown to develop a mutator phenotype that appears to drive the accumulation of mutations required for tumor development [42]. Therefore, we focused on the MMR genes among the DNA repair genes that were upregulated following infection (Supplementary Table S1). Among all MMR genes, we selected seven genes (*EXO1*, *UNG*, *PCNA*, *MSH2*, *MSH3*, *MSH6*, and *PMS2*) with increased expression levels after infection, as these genes cover the entire repair process from recognizing mismatch sites in infected cells to their ultimate repair.

### 2.2. Validation of the Increased Expression of the Seven MMR Genes at the mRNA Level in BVL-Infected Cattle in Vivo

To verify whether the expression levels of the seven MMR genes identified to be altered via NGS are increased at the mRNA level, we first performed a real-time quantitative reverse transcription–polymerase chain reaction (qRT-PCR) using in vitro infection models. RNA samples were extracted from Madin-Darby bovine kidney (MDBK), HeLa, pig kidney-15 (PK15), and Tb1Lu cells and then reverse-transcribed into cDNA. We used 0.5, 1.0, 2.0, and 4.0 ng of cDNA as a template to amplify the target MMR genes. The amplification efficiency of each primer (Supplementary Tables S2–S5) was based on the property of PCR in which the original DNA amount is doubled in one cycle. Relative expression levels were assessed based on the rise value (i.e., cycle threshold (C_T_) value) and the number of cycles of qRT-PCR using a two-fold dilution gradient of 0.5 to 4.0 ng. Measurements were then made using the slope and correlation coefficient (*R*^2^) value from the graph. Glyceraldehyde 3-phosphate dehydrogenase (*GAPDH*) was selected as the endogenous control gene for normalization. The amplification efficiency of each gene was achieved using a 7500 Fast Real-Time PCR system (Applied Biosystems, Foster City, CA, USA) as per the manufacturer’s instructions, within ±10% of the amplification efficiency value relative to that of *GAPDH* and an *R*^2^ value of more than 0.98.

**Figure 2:**
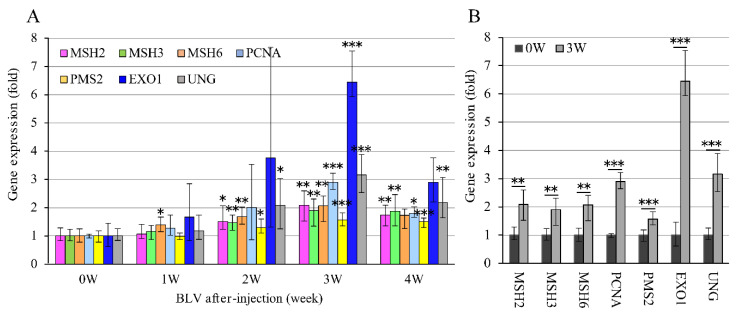
Comparison of expression levels at RNA level of 7 MMR genes in five calves before and after experimental BLV infection. (**A**) MMR gene expression at each time point in five BLV experimentally infected calves. Five BLV-negative calves carrying susceptible alleles BoLA-DRB3*1601/*1601 were experimentally challenged intravenously with BLV. RNAs were extracted from these calves at five time points in BLV post-infection (before BLV infection: 0 week; after BLV infection: 1–4 weeks), and then synthesized into cDNAs. The expression level of each MMR gene was measured by qRT-PCR. The expression level of MMR genes in each week was calculated by the expression of each gene before BLV infection (week 0), which was set as 1. *GAPDH* gene was used as an endogenous control for each gene expression analysis using the comparative C_T_ (∆∆C_T_) method. The vertical axis shows the expression level of each MMR gene, and the horizontal axis shows post-infection period. Simultaneously, BLV proviral loads were monitored at each time point by BLV-CoCoMo-qPCR-2 using genomic DNA that were extracted from the blood of five infected-calves at same points. (**B**) Of (**A**), only 0 week and 3 weeks at which expression mostly increased were shown. Expression was significantly increased in all MMR genes when compared within 0 week. *GAPDH* gene was used as an endogenous control for each gene expression analysis using the comparative C_T_ (∆∆C_T_) method. Error bars represent the standard deviation of three experiments. The *p* value was calculated by Student’s *t-*test. Asterisks indicate a significant difference (* *p* < 0.05, ** *p* < 0.01 and *** *p* < 0.001).

For in vivo validation, the expression levels of the seven MMR genes were also checked using qRT-PCR in RNA extracted from the blood of the same five calves before BLV infection (defined as week 0; individual negative control) and at different time points (weeks 1, 2, 3, and 4) after infection. The expression level of each gene at week 0 was assigned a value of 1 to calculate the relative expression levels of the seven genes at weeks 0–4 (Figure 2A). The BLV PVL was also evaluated at each time point by the BLV-CoCoMo-qPCR-2 method. At week 0, the PVL was 0 copies/10^5^ cells, which increased to 1158, 26,760, and 90,150 copies/10^5^ cells at weeks 1, 2, and 3, respectively, and then decreased to 62,440 copies/10^5^ cells by week 4. When calves were infected with BLV, the virus started infecting new cells, resulting in the small amounts of PVL in vivo at weeks 1. The only *MSH6* gene expression in week 1 was significantly higher than in week 0. The other five *MMR* gene expressions except for *PCNA* and *EXO1*, due to high variation, were significantly increased in week 2. Meanwhile, these seven gene expresses were increased at week 3, with the peak of PVL (Figure 2B). Although MMR expression started to decrease in week 4, a positive correlation between the upregulation of MMR genes and the BLV PVL was observed. Thus, these qRT-PCR data confirmed the NGS results that the expression levels of all studied MMR genes increased.

### 2.3. MMR Gene Expression is Upregulated in BLV-Infected Cattle with a High PVL in Vivo

**Figure 3:**
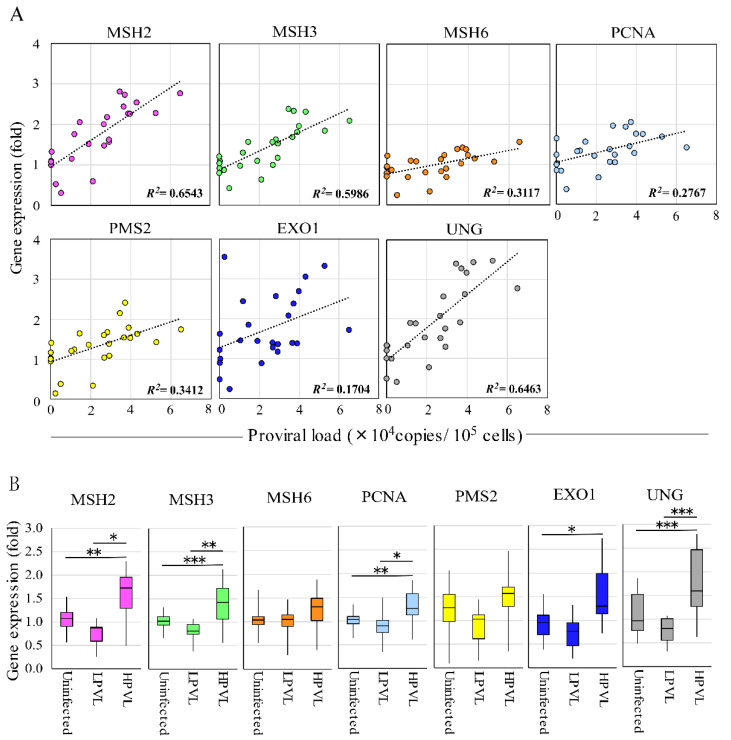
Correlation of expression of 7 MMR genes and proviral load in BLV-infected cattle in vivo. (**A**) The correlation between each MMR gene expression and proviral load (PVL). RNAs were extracted from 25 BLV-infected cattle to analyze MMR gene expression, and the fold-change in MMR gene expression was calculated using the ΔΔC_T_ method with normalization to *GAPDH* expression as an internal control. Spearman *r* was used to evaluate the strength of the correlation. (**B**) MMR gene expression in BLV-infected cattle with HPVL and LPVL. Cattle were divided into three groups: 20 BLV-negative cattle (proviral load = 0), 6 BLV-infected cattle with low-proviral loads (LPVL group = PVL < 10,000 copies/10^5^ cells), and 19 BLV-infected cattle with high-proviral loads (HPVL group = PVL ≥ 10,000 copies/10^5^ cells), and were measured for RNA expression levels of 7 MMR genes by qRT-PCR, and the fold-change was calculated. Samples were run in duplicate. The vertical axis indicates the relative expression of each MMR gene when the expression level of each gene in uninfected cattle was set to 1. The upper bar indicates maximum and lower bar shows the minimum values. The black line at the center of the graph indicates the median value. The *p* value was calculated by Tukey’s test after the analysis of variance. Asterisks indicate a significant difference (* *p* < 0.05, ** *p* < 0.01 and *** *p* < 0.001).

To analyze the correlation between the expression level of each MMR gene and the BLV PVL, we used 25 RNA and DNA samples extracted from field cattle infected with BLV using qRT-PCR and BLV-CoCoMo-qPCR-2 [32,43,44], respectively. The *R*^2^ value demonstrated a positive correlation between *MSH2* (0.6543), *MSH3* (0.5986), and *UNG* (0.6463) with PVLs. Moreover, *MSH6* (0.3117), *PCNA* (0.2767), and *PMS2* (0.3412) showed a weak correlation with PVLs (Figure 3A). In contrast, no correlation with PVL was observed for the *EXO1* gene. Next, we classified the cattle into three groups based on the PVL: BLV-negative cattle (control group), BLV-infected cattle with a low PVL (LPVL group), and BLV-infected cattle with a high PVL (HPVL group). As described previously, when the BLV PVL in the blood samples exceeded 10,000 copies/10^5^ cells, a high PVL was also detected in the nasal mucus, saliva [38], and milk sample [37] from BLV-infected cattle; therefore, a BLV PVL of 10,000 copies/10^5^ cells was used as the threshold to distinguish between the HPVL and LPVL groups [35]. As shown in Figure 3B, four of the seven MMR genes, including *MSH2*, *MSH3*, *PCNA*, and *UNG*, showed significantly upregulated expression in cattle of the HPVL group, but not in cattle of the LPVL group or uninfected cattle. Interestingly, *EXO1* showed significantly higher expression levels in cattle of the HPVL group than in uninfected cattle. The remaining two genes, *MSH6* and *PMS2*, also tended to be upregulated in cattle in the HPVL group. DNA damage tends to accumulate at genetic loci involved in the antiviral and stress response pathways that are highly upregulated after viral infection [45]. In addition, DNA MMR pathways are generally downregulated in cells infected with various RNA viruses [46,47]. These data strongly suggested that MMR genes are upregulated according to an increase in the PVL.

### 2.4. Analysis of MMR Gene Expression in BLV Transiently Transfected Cells and BLV Stably Expressing Cells

**Figure 4:**
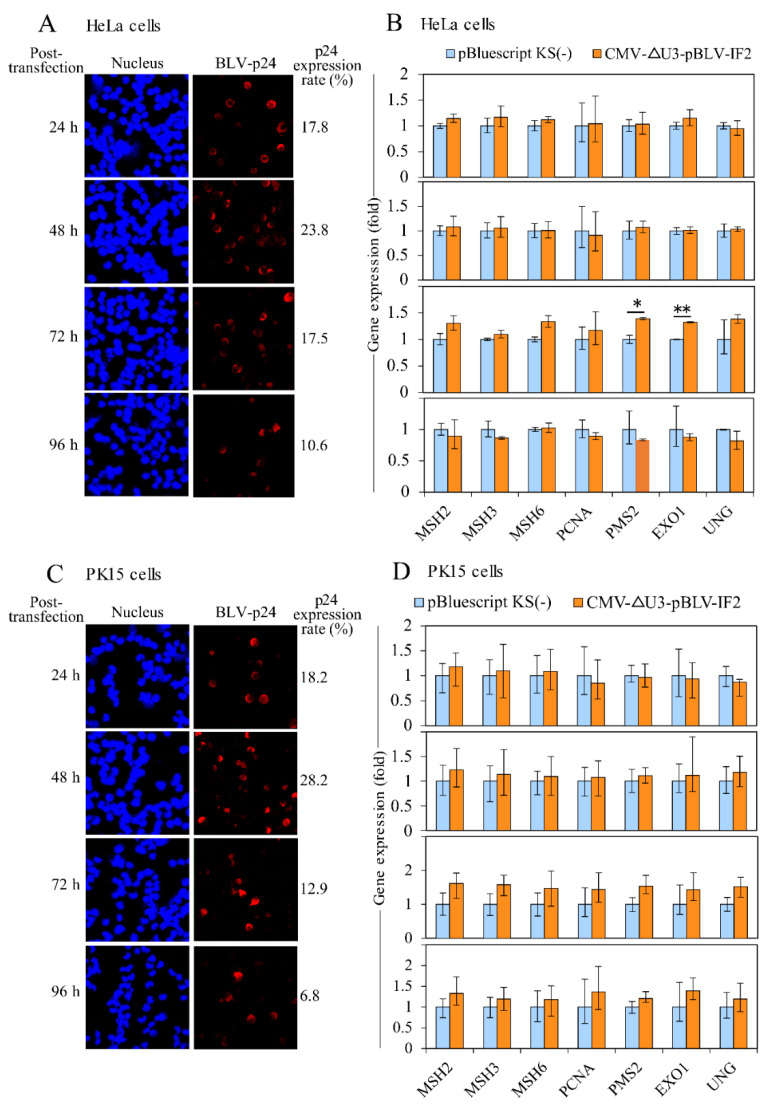
The expression of BLV and each MMR gene at transient levels in HeLa and PK15 cells with CMV-∆U3-pBLV-IF2. (**A**) The p24 expression in HeLa cells transfected with CMV-∆U3-pBLV-IF2. (**B**) The expression of each MMR gene in HeLa cells transfected CMV-∆U3-pBLV-IF2. (**C**) The p24 expression in PK15 cells transfected with CMV-∆U3-pBLV-IF2. (**D**) The expression of each MMR gene in PK15 cells transfected CMV-∆U3-pBLV-IF2. HeLa and PK15 cells were transfected with CMV-∆U3-pBLV-IF2 for 24, 48, 72, and 96 h; the expression rates of p24 were evaluated by a confocal laser microscope. The p24 expression rates were calculated by dividing the number of p24 expressing cells by the number of cells. The expressions of MMR gene were measured using qRT-PCR assay. Quantification was carried out using the ∆∆C_T_ method with *GAPDH* as the endogenous control. The vertical axis indicates the relative expression of each MMR gene when the expression level of each gene in cells transfected with pBluscript KS(−) was set to 1. The *MMR* gene expressions were observed to be higher in transfected cells with CMV-∆U3-pBLV-IF comparing with those in transfected cells with pBluscript KS(−) at 72 h. Error bars represent the standard deviation of three experiments. The *p* value was calculated by Student’s *t-*test. Significant differences are indicated by asterisks (* *p* < 0.05, and ** *p* < 0.01).

The data shown in Figure 3 indicate that the expression levels of MMR genes correlated well with the PVL in the context of BLV infection. To analyze the changes in MMR gene expression relative to the transient expression of BLV proteins in vitro, HeLa and PK15 cells were transfected with the BLV infectious molecular clone CMV-ΔU3-pBLV-IF2, and then BLV p24 was detected using an immunofluorescence assay at 24, 48, 72, and 96 h after transfection. The p24 expression ratio was calculated by dividing the number of p24-expressing cells by the total number of cells. After 48 h of transfection, 23.8% and 28.2% of the HeLa and PK15 cells, respectively, showed p24 expression (Figure 4A,C). Moreover, RNA was extracted from the transfected cells to evaluate the MMR gene expression using qRT-PCR. The relative expression level of each gene was normalized to the level of pBluescript KS(−) as a control, which was set to 1. The expression levels of MMR genes were the highest at 72 h after transfection in both HeLa and PK15 cells (Figure 4B,D). Specifically, the expression levels of *PMS2* and *EXO1* in HeLa cells were significantly increased compared with those of the control by 1.39 and 1.32 times, respectively. Increased expression levels of all MMR genes (*MSH2*: 1.61 times; *MSH3*: 1.58 times; *MSH6*: 1.46 times; *PCNA*: 1.44 times; *PMS2*: 1.53 times; *EXO1*: 1.43 times; *UNG*: 1.52 times) were observed at 72 h after transfection in PK15 cells. 

**Figure 5:**
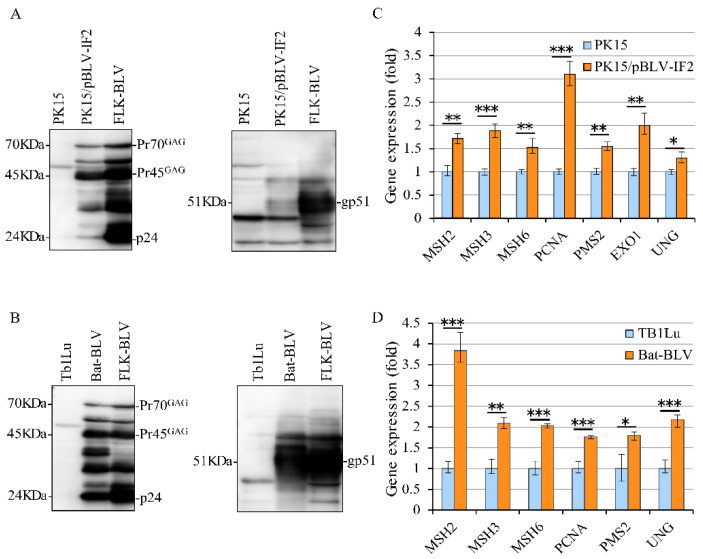
Comparison of MMR expression levels in PK15/pBLV-IF2 and Bat-BLV cells. (**A**) BLV expression in the parental PK15 and PK15/pBLV-IF2 cells, and (**B**) parental Tb1Lu and Bat-BLV. After 48 h cultured, PK15/pBLV-IF2 and parental PK15 cells, and Bat-BLV and parental Tb1Lu cells were harvested, lysis, and then used to detect viral proteins expression with anti-gp51 antibody and anti-p24 antibody by Western blot. Positions of BLV p24, Pr45^GAG^, Pr70^GAG^ and gp51 proteins, and molecular masses are indicated. (**C**) MMR gene expressions in PK15 and PK15/pBLV-IF2 cells, and (**D**) Tb1Lu and Bat-BLV. The cDNAs were synthesized to evaluate the expression level of the MMR gene using qRT-PCR. *EXO1* was not identified in bat cells. The vertical axis represents the relative expression level when the expression level of each gene in parental cells was set to 1. Error bars represent the standard deviation of three experiments. The *p* value was calculated by Student’s *t-*test. Significant differences are indicated by asterisks (* *p* < 0.05, ** *p* < 0.01, and *** *p* < 0.001).

To confirm the increased expression levels of MMR genes at a transient level in both HeLa and PK15 cells, we used BLV-expressing PK15/pBLV-IF2 and Bat-BLV cells and their parental uninfected cell lines. Bat-BLV is a permanently BLV-infected cell line, and the parental Tb1Lu cells [48] and PK15/pBLV-IF2 were produced by our group 2 years ago as a stably transfected cell line with CMV-ΔU3-pBLV-IF2 [22,49]. We first confirmed the expression of BLV protein in the stably expressing PK15/pBLV-IF2 and Bat-BLV cell lines by Western blotting with an anti-p24 monoclonal antibody (Mab; BLV-3) and anti-gp51 Mab (BLV-2). Specific bands for p24, Pr70^GAG^, and Pr45^GAG^ were detected at positions 24, 45, and 70 kDa in both cell lines (Figure 5A,B). However, a weak band was detected at position 51 kDa, which was attributed to the anti-gp51 Mab in PK15/pBLV-IF2 cells (Figure 5A). This specific band of 51 kDa was also detected in Bat-BLV and FLK-BLV cells. Hence, we confirmed that BLV protein is stably expressed in both PK15/pBLV-IF2 and Bat-BLV cells. Therefore, we analyzed the MMR gene expression in PK15 and PK15/pBLV-IF cells as well as in their parental cell lines Tb1Lu and Bat-BLV. The expression levels of all tested MMR genes was significantly higher in PK15/pBLV-IF2 cells compared to those in PK15 cells (Figure 5C) and in Bat-BLV cells compared to those in Tb1Lu cells (Figure 5D), confirming the increased expression of MMR genes found at transient levels for BLV stably expressing cell lines. These results showed that BLV protein expression upregulates the expression of MMR genes in vitro. Other mutations in BLV stably expressing cell lines also need to be analyzed in detail to obtain a full picture of the gene expression changes in the future.

### 2.5. MMR Gene Expression is Downregulated in BLV-Infected Cattle with Lymphoma in Vivo

**Figure 6:**
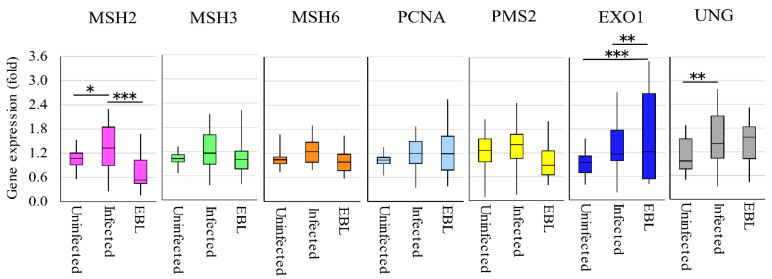
The expression levels of MMR genes in different cattle groups. DNAs were extracted from bloods of cattle containing 20 uninfected cattle, 25 BLV-infected without onset, and 20 EBL with lymphoma to measure the expression of MMR gene using qRT-PCR. The vertical axis indicates the relative expression of each MMR gene when the expression level of each gene in uninfected cattle was set to 1. The upper bar indicates maximum and lower bar shows the minimum values. The black line at the center of the graph indicates the median value. The *p* value was calculated by Tukey’s test after the analysis of variance. Significant differences are indicated by asterisks (* *p* < 0.05, ** *p* < 0.01 and *** *p* < 0.001).

We then investigated whether a change occurred in the expression of MMR genes in BLV-infected cattle with lymphoma. Briefly, RNA samples were extracted from BLV-negative cattle (*n* = 20), BLV-infected cattle without lymphoma (*n* = 25), and BLV-infected cattle with lymphoma (*n* = 20) to measure the gene expression levels of the seven MMR genes (Figure 6). The expression levels of *MSH2* was significantly lower in the lymphoma group than those in the BLV-infected cattle groups. Conversely, *MSH2* and *UNG* were expressed at significantly higher levels in infected cattle than those in uninfected cattle. *EXO1* was expressed at significantly higher levels in infected cattle with lymphoma than in uninfected cattle and BLV-infected cattle. In addition, the expression levels of *PMS2* tended to be lower in the lymphoma group than in the BLV-infected cattle groups. The levels of *MSH3*, *MSH6*, *PCNA*, and *UNG* did not show any difference between BLV-infected and EBL groups. Our results also indicated that the expression of *MSH2* and *UNG* is promoted in BLV-infected cattle, whereas the expression of *MSH2* is suppressed in BLV-infected cattle with lymphoma in vivo.

## 3. Discussion

Some BLV-infected individuals do not show changes in p53, TNF-α activity, and *BoLA-DRB3* allele frequency, suggesting that an additional host factor regulates the PVL in vivo. BLV may integrate within the genomic DNA of B-lymphocytes leading to the PVL or can exist as unintegrated circular or linear forms. MMR is a mechanism for recognizing and repairing erroneous insertions, deletions, and misincorporation of bases that can arise during DNA replication and recombination, as well as for repairing other forms of DNA damage. The following three main results were obtained in the present study. First, the expression of all seven MMR genes (i.e., *MSH2*, *MSH3*, *MSH6*, *PCNA*, *PMS2*, *EXO1*, and *UNG*) in BLV-experimentally infected calves, and the other five MMR genes except for *MSH6* and *PMS2* in naturally infected cattle with HPLV were upregulated in vivo. This suggests that MMR genes are activated by BLV infection. Second, the expression levels of all seven MMR genes showed an increasing tendency in BLV transiently transfected cells and were clearly increased in BLV stably expressing cells in vitro. This reveals that the expression levels of MMR genes increase in parallel with the expression levels of BLV proteins, rather than due to the effect of the transfection itself on the DNA repair mechanisms. The results of these in vivo and in vitro experiments suggest that BLV might improve the function of MMR by increasing the MMR gene expression levels during infection. Third, the expression levels of *MSH2* significantly decreased in BLV-infected cattle with lymphoma. In addition, *MSH6* and *PMS2* tended to be lower in BLV-infected cattle with lymphoma compared within infected cattle. *EXO1* was significantly increased in BLV-infected cattle with lymphoma compared with uninfected and infected cattle. These MMR proteins recognize and bind to the mismatch site; thus, when they are downregulated, the mismatch site cannot be correctly identified and bound. This results in an increase in incorrect sequences in vivo that might influence cell transformation and tumorigenesis. However, whether the expression of these four genes affects the transformation of CD5+ B cells, which are the target of tumorigenesis in individuals with lymphoma, remains unknown. Taken together, these results suggest that the expression levels of MMR genes increase to enhance MMR function during BLV infection and then decrease to attenuate the MMR function during the onset of EBL.

According to both the NGS and qRT-PCR results, the expression levels of MMR genes were found to increase in five cattle experimentally infected with BLV carrying a susceptible allele, BoLA-DRB3*1601, in homozygous form. In addition, MMR gene expression levels increased in BLV-infected field cattle with a HPVL. This strongly indicates that the expression of MMR genes increases with BLV infection. HTLV-1, a human virus related to BLV, encodes several regulatory and accessory proteins in addition to the transcriptional activator Tax that is involved in tumorigenesis. Several studies have clarified the function of Tax, including its association with DNA repair mechanisms [50,51,52]. Regarding the relationship between MMR and Tax, *PCNA*, which is a cofactor of DNA replication, is suppressed by the transitivity of Tax [53]. *PMS1* is a MMR gene whose expression is suppressed by methylation of the promoter region by Tax to attenuate the MMR process [54]. In addition, HBZ protein, which is transcribed from the 3′ terminus of the HTLV-1 genome and is constantly produced in HTLV-1-infected cells, inhibits the MMR process [55,56]. Another recent study showed that BLV encodes antisense protein and microRNA at the 3′ terminus, but their functions have not yet been elucidated. Although we did not perform any experiments using these BLV proteins, it is important to further analyze the expression of MMR genes using an expression vector incorporating each gene of BLV to elucidate the regulatory mechanism of MMR genes by BLV.

Analysis of the expression of MMR genes in cattle with EBL and uninfected cattle revealed a low *MSH2* expression level. As MSH2 is an essential protein for recognizing DNA mismatches, it is considered to be the most important protein for DNA repair in the MMR system [57,58,59]. Furthermore, MSH2 is considered to act as a tumor suppressor given its primary functions of cell cycle regulation [60,61,62], and the induction of apoptosis under excessive MSH2 protein expression [12,63]. According to several reports, it is possible that MSH2 protein plays a major role in regulating the concertation of CD5+ B cells in cattle with EBL. In the future, it will be particularly important to analyze the relationship between cell carcinogenesis and MMR expression at both the gene and protein levels.

In the present study, we identified MMR genes as host genes whose expression was affected by the change of PVL in BLV-infected cattle. However, MMR genes cannot serve as a surrogate for PVL, and these two markers provide different information. This is because when cattle are infected with BLV, the PVL in vivo gradually increases in a manner dependent on BLV disease progression, and remains high in cattle developing lymphoma [32,35]. By contrast, upon BLV infection, the expression levels of MMR gene increase in a manner dependent on the rate of BLV infecting new cells, but then decrease at the lymphoma stage, indicating that MMR genes may have little to no function at the lymphoma stage. In addition, our results demonstrate an important shift at the point when the MMR expression levels begin to decline. However, the precise time point at which MMR expression levels starts to decline when the infection progresses to lymphoma could not be determined based on the samples analyzed in this study. Further investigations will be needed in cattle with BLV infection at different stages of disease progression from before and after the onset of lymphoma.

MMR-deficient cells can display a mutator phenotype that is characterized by microsatellite instability and an elevated mutation frequency. In addition, germline mutations in MMR genes can lead to a variety of cancers [64]. According to the present results, when animals are infected with BLV, MMR genes would be activated depending on the increase in PVLs in BLV-infected cattle without lymphoma. Therefore, when BLV infects new cells, MMR gene expression will be initially upregulated to ensure survival of the host cell as the host DNA is constantly under assault from both exogenous and endogenous DNA-damaging agents. However, once the BLV-infected cells undergo malignant transformation, MMR genes are suppressed. We propose two hypotheses to explain these changes: malignant transformation-induced mutations or deletions occur to suppress MMR function, or loss of function occurs from factors in the tumor cells. Therefore, MMR genes were highly expressed in cattle with a HPVL compared with those in BLV-negative cattle and in BLV-infected cattle with a LPVL. Moreover, MMR gene expression levels tended to decrease in BLV-infected cattle with lymphoma compared with those in BLV-infected cattle without lymphoma in vivo. Therefore, MMR gene expression can serve as a useful indicator of the severity of BLV infection.

## 4. Materials and Methods

### 4.1. Blood Samples from Field Cattle and Cattle Experimentally Infected with BLV

Five Japanese Black cattle carrying a homozygous BoLA-DRB3*1601 allele were experimentally challenged by an intravenous injection of white blood cells containing a PVL of 4 × 10^7^ copies/10^5^ cells obtained from BLV-seropositive Holstein–Friesian cattle. Whole blood samples (in ethylenediaminetetraacetic acid (EDTA)) were collected every other week starting at one week before infection, for a total of five times. This study was approved by the Animal Ethical Committee, and the Animal Care and Use Committee of Kyoto Biken Institute. For field samples, blood samples were collected from 65 Holstein cattle, including BLV-negative cattle (*n* = 20), BLV-infected cattle without lymphoma (*n* = 25), and cattle with EBL that developed B-cell lymphoma (*n* = 20). This study was approved by the Animal Ethical Committee, and the Animal Care and Use RIKEN Animal Experiments Committee (approval number H29-2-104).

### 4.2. RNA and DNA Extraction

The same volume of Ambion nuclease-free water (Thermo Fisher Scientific, Tokyo, Japan) was added to all whole blood samples (in EDTA) for dilution, followed by mixing with a triplicate volume of TRIzol LS Reagent (Thermo Fisher Scientific, Tokyo, Japan); the samples were stored at −80 °C until use. RNA was then extracted from the mixture of TRIzol LS Reagent and whole blood according to the manufacturer’s instructions. Briefly, we added 0.2 mL of chloroform into 0.75 mL of the mixture of TRIzol LS Reagent and whole blood samples for lysis. After centrifugation for 10 min at 12,000× *g* and 4 °C, the supernatant was discarded, and the pellet was resuspended in 1 mL of 75% ethanol to centrifuge again. After removing the supernatant, the sample was air-dried and 20–50 µL of Ambion Nuclease-Free Water was added. DNA was extracted from the blood samples using Wizard Genomic DNA Purification Kit (Promega Corp., Tokyo, Japan) at each time point to measure the PVL using the BLV-CoCoMo-qPCR-2 method [32,43,44].

### 4.3. Construction of the RNA Library

RNA was first mixed with Bibo-Zero reaction buffer and rRNA Removal agent and then heat-treated at 68 °C for 10 min to remove ribosomes. The RNA samples were then purified using Agencourt RNAClean XP beads (Beckman Coulter, Indianapolis, IN, USA). The RNA library was constructed using the TruSeq Stranded Total RNA with Ribo-Zero Globin kit (Illumina, San Diego, CA, USA) according to the manufacturer’s instructions. 

The purified RNA was then fragmented using a T100 thermal cycler (Bio-Rad Laboratories, Inc., Tokyo, Japan) at 95 °C for 8 min and at 4 °C for 5 min. This fragmented RNA was then mixed with First Strand Mix (Illumina, San Diego, CA, USA) and ProtoScript II Reverse Transcriptase (BioLabs, Tokyo, Japan) and was reacted as follows: 25 °C for 10 min, 42 °C for 50 min, and 70 °C for 15 min. Next, Second Strand Mix (Illumina, San Diego, CA, USA) was added to the reaction mixtures, and cDNA was synthesized at 16 °C for 60 min.

All cDNA samples were then purified using Agencourt RNAClean XP beads (Beckman Coulter, Indianapolis, IN, USA), End Repair Mix (New England BioLabs, Ipswich, MA, USA) was added to the purified cDNA to perform a 5′-end repair reaction, followed by mixing with A-Tailing Mix (New England BioLabs, Ipswich, MA, USA) to add adenyl groups at the 3′ end. Adaptor Index was then added to the samples for the adaptor binding reaction, followed by another purification step using AMPure XP beads (Beckman Coulter, Indianapolis, IN, USA). Finally, PCR Primer Cocktail and PCR Master Mix (Illumina, San Diego, CA, USA) were added to the cDNA samples to perform PCR. 

### 4.4. Analysis of Gene Expression by NGS

Deep sequencing was performed using an Illumina Hiseq2500 platform (Illumina, San Diego, CA, USA). Sequence reads were mapped to Bostau6 (UMD3.1) bovine genome builds using TopHat [65]. The numbers of sequence read mapped to each exon were counted using the hiseq-count program (https://htseq.readthedocs.io/en/master/). The data count was analyzed using DESeq [66] to calculate the normalized fold change between the sample collected before BLV infection (0-week group) and after BLV infection (3-week group). The obtained gene list was analyzed using the free gene expression analysis tool PANTHER (http://pantherdb.org/) and subjected to GO functional annotation. 

### 4.5. qRT-PCR

Extracted RNA was first reverse-transcribed into cDNA using the High-Capacity RNA-to-cDNA kit (Thermo Fisher Scientific, Waltham, MA, USA) according to the manufacturer’s instructions. After cDNA synthesis, KAPA SYBR FAST qPCR Kits (Kapa Biosystems, Wilmington, MA, USA) were used to perform qRT-PCR. We used the genome information of cattle, humans, and bats from the National Center for Biotechnology Information (http://www.ncbi.nlm.nih.gov/) as reference DNA for primer design and used Primer3 Input (ver. 0.4.0) software (http://bioinfo.ut.ee/primer3-0.4.0/) to construct primers (Supplementary Tables S2–S5). *GAPDH* was used as an endogenous control for each gene expression analysis using the comparative C_T_ (∆∆C_T_) method.

### 4.6. Cell Culture and Transfection

Human cervical HeLa cells, permanently BLV-infected fetal lamb cell line FLK-BLV, bat lung cell line Tb1Lu, and permanently BLV-infected bat cell line Bat-BLV were cultured in Dulbecco’s modified Eagle medium (Thermo Fisher Scientific, Waltham, MA, USA) supplemented with 10% fetal bovine serum (FBS; MilliporeSigma, Burlington, MA, USA) at 37 °C with 5% CO_2_. The PK15 cell line was cultured in Eagle’s minimum essential medium (Thermo Fisher Scientific) containing 10% FBS.

Hela cells (1.0 × 10^5^/well), Tb1Lu cells (1.0 × 10^5^/well), and PK15 cells (8.0 × 10^4^/well) were seeded on a 12-well plate for 20 h and then co-transfected with 2 µg of CMVΔU3-pBLV-IF2 and 0.2 µg of pEGFP-N1 [67] or a negative control vector (pBluescript II KS(−); Stratagene, La Jolla, CA, USA). CMVΔU3-pBLV-IF2 is a modified infectious molecular clone of pBLV-IF [48,68,69,70], with a cytomegalovirus (CMV) promoter instead of the U3 region at the 5′ terminus of the long terminal repeat. 

### 4.7. Measurement of the BLV PVL

The BLV PVL in all DNA samples was measured using the BLV-CoCoMo-qPCR-2 method (RIKEN Genesis, Kanagawa, Japan) as previously described [32,34,38,43,44,71,72].

### 4.8. Typing of the BoLA-DRB3 Allele

cDNA was amplified by PCR using primers F (5′-CGCTCCTGTGAYCAGATCTATCC-3′) and R (5′-CACCCCCGCGCTCACC-3′). All PCR products were purified and sequenced using BigDye Terminator v1.1 Cycle Sequencing Kit (Thermo Fisher Scientific, Waltham, MA, USA). All sequencing data were analyzed using Assign 400ATF software (Conexio Genomics, Fremantle, Australia).

### 4.9. Western Blotting Analysis

Cell lysates of the PK15, PK15/pBLV-IF2, TB1Lu, and Bat-BLV lines were prepared using a lysis buffer containing 10 mM Tris-HCl (pH 8.0), 150 mM NaCl, 1 mM EDTA, 1% NP-40, and protease inhibitor (MilliporeSigma, Burlington, MA, USA), followed by quantification using a bicinchoninic acid protein assay kit (Thermo Fisher Scientific, Waltham, MA, USA). Western blotting was then performed with an anti-gp51 Mab (BLV-2; VMRD, Pullman, WA, USA) or an anti-BLV Gag protein Mab (BLV-3; VMRD), followed by incubation with horseradish peroxidase-conjugated goat anti-mouse IgG (Amersham Biosciences, Piscataway, NJ, USA). 

### 4.10. Immunofluorescence Assay

For cell fixing, microslides (Matsunami, Osaka, Japan) seeded with transfected HeLa and Tb1Lu1 cells were treated with methanol (Sigma-Aldrich, St. Louis, MO, USA) at −20 °C for 15 min. After washing, anti-gp51 Mab (BLV-1; VMRD, Pullman, WA, USA) or anti-Gag Mab (BLV-3; VMRD) was added to the slides to react, followed by Alexa Fluor 594 goat anti-mouse IgG (H + L; Thermo Fisher Scientific, Waltham, MA, USA). The nuclei were then stained with Hoechst 33342 (Sigma-Aldrich, St. Louis, MO, USA) in the dark. The fluorescence of the Alexa Fluor 594 in the cells was visualized and analyzed using an FV1000 confocal laser-scanning microscope (Olympus, Tokyo, Japan).

### 4.11. Statistical Analysis

All data of in vitro experiments are expressed as the mean ± standard deviation based on at least three independent experiments. A Student’s *t*-test was used to calculate the significance of the differences in the means of MMR gene expression levels between two samples. Analysis of variance followed by Tukey’s test for post-hoc analysis was used to determine the significance of the means of MMR gene expression levels for multiple comparisons. Differences were considered to be significant at *p* < 0.05, and strongly significant at *p* < 0.01 and *p* < 0.001.

## 5. Conclusions

The results obtained in this study indicate that BLV infection affects MMR gene expression in vivo (i.e., in experimentally infected cattle and field-infected cattle) and in vitro (i.e., in BLV transient expression cells and BLV stable expression cells). All seven MMR genes were upregulated in five BLV-experimental infected calves. The expressions of *MSH2*, *MSH3*, *PCNA*, *EXO1*, and *UNG* were upregulated in cattle with a HPVL compared with those in cattle from the negative control group. Expressions of *MSH2* was downregulated in cattle with EBL compared with it in BLV-infected cattle. In addition, we found that the expression levels of *MSH6* and *PMS2* were lower in cattle with EBL compared with those in BLV-infected cattle. EXO1 was significantly increased in BLV-infected cattle with lymphoma compared within uninfected cattle and BLV-infected cattle without lymphoma. It is important to investigate the specific role of MSH2 in BLV infection—i.e., if it prevents mutations or regulates recombination at dissimilar sequences. Furthermore, the relationship between *EXO1* and innate antiviral response, which together control the severity of BLV infection, warrants further investigation. In the future, it will be important to identify the viral factor of BLV that influences MMR and to elucidate its regulatory mechanism.

## Figures and Tables

**Table 1:** Top ten upregulated biological functions after bovine leukemia virus (BLV) infection.

Biological Process (Upregulate)	*p*-Value	FDR	Significance
DNA metabolic process	1.01 × 10^−25^	2.45 × 10^−23^	24.996
nitrogen compound metabolic process	3.32 × 10^−21^	4.05 × 10^−19^	20.479
nucleobase-containing compound metabolic	1.16 × 10^−20^	9.46 × 10^−19^	19.936
cell cycle	2.28 × 10^−17^	1.39 × 10^−15^	16.642
metabolic process	3.77 × 10^−17^	1.84 × 10^−15^	16.424
primary metabolic process	3.87 × 10^−16^	1.58 × 10^−14^	15.412
DNA repair	8.74 × 10^−16^	3.05 × 10^−14^	15.058
DNA replication	1.32 × 10^−14^	4.03 × 10^−13^	13.879
organelle organization	3.50 × 10^−12^	9.49 × 10^−11^	11.456
mitosis	7.58 × 10^−11^	1.85 × 10^−9^	10.12

## Supplementary Materials

The following are available online at https://www.mdpi.com/2076-0817/9/11/909/s1, Table S1: The List of DNA repair genes in upregulated genes from NGS result; Table S2: Real-time PCR primers targeting bovine MMR genes; Table S3: Real-time PCR primers targeting human MMR genes; Table S4: Real-time PCR primers targeting porcine MMR genes; Table S5: Real-time PCR primers targeting bat MMR genes.
