# Supplementary material for: Bovine Leukemia Virus Infection Affects Host Gene Expression Associated with DNA Mismatch Repair
Source: Pathogens. 2020 Oct 30;9(11):909. doi: 10.3390/pathogens9110909 (PMC7694100; doi:10.3390/pathogens9110909)
Supplement: Supplementary file 1 [file pathogens-09-00909-s001.pdf]

Supplementary data

Table S1. The List of DNA repair genes in upregulated genes from NGS result.

	Mapped ID	gene symbol	Gene	Panther class information
1	BOVIN|Gene=APEX1|UniProtKB=A0A140T846	APEX1	DNA-(apurinic or apyrimidinic site) lyase;APEX1;ortholog	
2	BOVIN|Ensembl=ENSBTAG00000003111|UniProtKB=E1BEI6	ATM	Serine-protein kinase ATM;ATM;ortholog	non-receptor serine/threonine protein kinase(PC00167)
3	BOVIN|Gene=BCCIP|UniProtKB=Q2NL37	BCCIP	BRCA2 and CDKN1A-interacting protein;BCCIP;ortholog	
4	BOVIN|Ensembl=ENSBTAG00000020301|UniProtKB=E1BQ04	BLM	BLM RecQ like helicase;BLM;ortholog	DNA helicase(PC00011)
5	BOVIN|Gene=BRCA1|UniProtKB=F1MYX8	BRCA1	Breast cancer type 1 susceptibility protein homolog;BRCA1;ortholog	ubiquitin-protein ligase(PC00234)
6	BOVIN|Gene=BRCA2|UniProtKB=E1B8X8	BRCA2	BRCA2, DNA repair associated;BRCA2;ortholog	damaged DNA-binding protein(PC00086)
7	BOVIN|Ensembl=ENSBTAG00000012068|UniProtKB=F1MMS0	BRIP1	BRCA1 interacting protein C-terminal helicase 1;BRIP1;ortholog	DNA helicase(PC00011)
8	BOVIN|Gene=CHAF1A|UniProtKB=A6QLA6	CHAF1A	Chromatin assembly factor 1 subunit A;CHAF1A;ortholog	chromatin/chromatin-binding, or -regulatory protein(PC00077)
9	BOVIN|Gene=CHEK1|UniProtKB=F6Q6K1	CHEK1	Checkpoint kinase 1;CHEK1;ortholog	non-receptor serine/threonine protein kinase(PC00167)
10	BOVIN|Ensembl=ENSBTAG00000020193|UniProtKB=E1BN11	DCLRE1A	DNA cross-link repair 1A;DCLRE1A;ortholog	
11	BOVIN|Gene=DNA2|UniProtKB=E1BMP7	DNA2	DNA replication ATP-dependent helicase/nuclease DNA2;DNA2;ortholog	RNA helicase(PC00032);DNA helicase(PC00011)
12	BOVIN|Gene=DTL|UniProtKB=E1BJV8	DTL	Denticleless E3 ubiquitin protein ligase homolog;DTL;ortholog	
13	BOVIN|Ensembl=ENSBTAG00000009396|UniProtKB=E1BF15	EXO1	Uncharacterized protein;EXO1;ortholog	exodeoxyribonuclease(PC00098)
14	BOVIN|Gene=FANCC|UniProtKB=O19104	FANCC	Fanconi anemia group C protein homolog;FANCC;ortholog	
15	BOVIN|Ensembl=ENSBTAG00000010077|UniProtKB=E1BE83	FANCD2	Uncharacterized protein;FANCD2;ortholog	
16	BOVIN|Gene=FANCI|UniProtKB=E1BDI4	FANCI	Uncharacterized protein;FANCI;ortholog	

17	BOVIN|Ensembl=ENSBTAG00000002612|UniProtKB=A0A3Q1MT39	FANCM	Uncharacterized protein;FANCM;ortholog	DNA binding protein(PC00009)
18	BOVIN|Gene=GEN1|UniProtKB=E1B8D0	GEN1	Uncharacterized protein;GEN1;ortholog	exodeoxyribonuclease(PC00098)
19	BOVIN|Ensembl=ENSBTAG00000044006|UniProtKB=E1BC42	GINS2	DNA replication complex GINS protein PSF2;GINS2;ortholog	DNA binding protein(PC00009)
20	BOVIN|Ensembl=ENSBTAG00000017133|UniProtKB=A2VE40	GINS4	DNA replication complex GINS protein SLD5;GINS4;ortholog	
21	BOVIN|Ensembl=ENSBTAG00000018151|UniProtKB=A0A3Q1LQC3	HELQ	Helicase, POLQ like;HELQ;ortholog	
22	BOVIN|Ensembl=ENSBTAG00000034529|UniProtKB=A0A3Q1N116	HMGA1	Uncharacterized protein;HMGA1;ortholog	endodeoxyribonuclease(PC00093)
23	BOVIN|Gene=KIF22|UniProtKB=A6QPL4	KIF22	Kinesin-like protein KIF22;KIF22;ortholog	microtubule binding motor protein(PC00156)
24	BOVIN|Ensembl=ENSBTAG00000014595|UniProtKB=A0A3S5ZPU0	LIG1	DNA ligase;LIG1;ortholog	DNA ligase(PC00012)
25	BOVIN|Ensembl=ENSBTAG00000014380|UniProtKB=A0A3Q1LHC9	MCM2	DNA helicase;MCM2;ortholog	DNA binding protein(PC00009)
26	BOVIN|Gene=MCM3|UniProtKB=A4FUD9	MCM3	DNA replication licensing factor MCM3;MCM3;ortholog	DNA binding protein(PC00009)
27	BOVIN|Ensembl=ENSBTAG00000017021|UniProtKB=E1BFZ9	MCM4	DNA helicase;MCM4;ortholog	DNA binding protein(PC00009)
28	BOVIN|Gene=MCM5|UniProtKB=A6H7F8	MCM5	DNA helicase;MCM5;ortholog	DNA binding protein(PC00009)
29	BOVIN|Gene=MCM6|UniProtKB=E1BH89	MCM6	DNA helicase;MCM6;ortholog	DNA binding protein(PC00009)
30	BOVIN|Gene=MCM7|UniProtKB=Q3ZBH9	MCM7	DNA replication licensing factor MCM7;MCM7;ortholog	DNA binding protein(PC00009)
31	BOVIN|Ensembl=ENSBTAG00000003425|UniProtKB=A0A3Q1M4B9	MGME1	Mitochondrial genome maintenance exonuclease 1;MGME1;ortholog	
32	BOVIN|Ensembl=ENSBTAG00000000629|UniProtKB=A0A3Q1LTN5	MMS22L	Protein MMS22-like;MMS22L;ortholog	
33	BOVIN|Gene=MMS22L|UniProtKB=E1BGH8	MMS22L	Protein MMS22-like;MMS22L;ortholog	
34	BOVIN|Ensembl=ENSBTAG00000002742|UniProtKB=Q3MHE4	MSH2	DNA mismatch repair protein Msh2;MSH2;ortholog	DNA binding protein(PC00009)
35	BOVIN|Ensembl=ENSBTAG00000007684|UniProtKB=A0A3Q1LUI6	MSH3	DNA mismatch repair protein;MSH3;ortholog	DNA binding protein(PC00009)

36	BOVIN|Ensembl=ENSBTAG00000001424|UniProtKB=E1B9Q4	MSH6	Uncharacterized protein;MSH6;ortholog	DNA binding protein(PC00009)
37	BOVIN|Ensembl=ENSBTAG00000011242|UniProtKB=F1N4K4	MUTYH	Adenine DNA glycosylase;MUTYH;ortholog	DNA glycosylase(PC00010)
38	BOVIN|Gene=NPM1|UniProtKB=Q3T160	NPM1	Nucleophosmin;NPM1;ortholog	chaperone(PC00072)
39	BOVIN|Ensembl=ENSBTAG00000007777|UniProtKB=F1MPV2	OGG1	Uncharacterized protein;OGG1;ortholog	
40	BOVIN|Ensembl=ENSBTAG00000006405|UniProtKB=E1BL90	PALB2	Uncharacterized protein;PALB2;ortholog	
41	BOVIN|Gene=PARP1|UniProtKB=P18493	PARP1	Poly [ADP-ribose] polymerase 1;PARP1;ortholog	
42	BOVIN|Ensembl=ENSBTAG00000033983|UniProtKB=Q0VD16	PCNA	Proliferating cell nuclear antigen	
43	BOVIN|Ensembl=ENSBTAG00000007364|UniProtKB=A0A3Q1M1S8	PMS2	Uncharacterized protein;PMS2;ortholog	DNA binding protein(PC00009)
44	BOVIN|Gene=POLD1|UniProtKB=P28339	POLD1	DNA polymerase delta catalytic subunit;POLD1;ortholog	
45	BOVIN|Ensembl=ENSBTAG00000011083|UniProtKB=E1BNZ6	POLD1	DNA polymerase;POLD1;ortholog	
46	BOVIN|Ensembl=ENSBTAG00000000590|UniProtKB=E1BIF0	POLE	DNA polymerase epsilon catalytic subunit;POLE;ortholog	
47	BOVIN|Gene=POLE2|UniProtKB=A7YWS7	POLE2	DNA polymerase epsilon subunit 2;POLE2;ortholog	DNA-directed DNA polymerase(PC00018)
48	BOVIN|Ensembl=ENSBTAG00000006015|UniProtKB=F1MZ79	POLH	Uncharacterized protein;POLH;ortholog	
49	BOVIN|Gene=POLQ|UniProtKB=E1BMC0	POLQ	DNA polymerase theta;POLQ;ortholog	DNA-directed DNA polymerase(PC00018)
50	BOVIN|Ensembl=ENSBTAG00000017019|UniProtKB=E1BLB6	PRKDC	Uncharacterized protein;PRKDC;ortholog	non-receptor serine/threonine protein kinase(PC00167)
51	BOVIN|Gene=PRPF19|UniProtKB=Q08E38	PRPF19	Pre-mRNA-processing factor 19;PRPF19;ortholog	
52	BOVIN|Ensembl=ENSBTAG00000012142|UniProtKB=A0A3Q1M8J6	RAD18	Uncharacterized protein;RAD18;ortholog	ubiquitin-protein ligase(PC00234)
53	BOVIN|Ensembl=ENSBTAG00000011252|UniProtKB=G3X6W2	RAD50	Uncharacterized protein;RAD50;ortholog	

54	BOVIN|Gene=RAD51|UniProtKB=Q2KJ94	RAD51	DNA repair protein RAD51 homolog 1;RAD51;ortholog	
55	BOVIN|Ensembl=ENSBTAG00000015036|UniProtKB=F1MW99	RAD51C	RAD51 paralog C;RAD51C;ortholog	
56	BOVIN|Gene=RAD54L|UniProtKB=A1L4Z4	RAD54L	RAD54 like;RAD54L;ortholog	
57	BOVIN|Gene=RFC2|UniProtKB=Q05B83	RFC2	Replication factor C subunit 2;RFC2;ortholog	DNA-directed DNA polymerase(PC00018)
58	BOVIN|Ensembl=ENSBTAG00000010787|UniProtKB=Q2TBV1	RFC3	Replication factor C subunit 3;RFC3;ortholog	DNA-directed DNA polymerase(PC00018)
59	BOVIN|Ensembl=ENSBTAG00000004460|UniProtKB=F1MAV6	RFWD3	Ring finger and WD repeat domain 3;RFWD3;ortholog	ubiquitin-protein ligase(PC00234)
60	BOVIN|Ensembl=ENSBTAG00000015878|UniProtKB=Q1LZE2	RHNO1	RAD9, HUS1, RAD1-interacting nuclear orphan protein 1;RHNO1;ortholog	
61	BOVIN|Ensembl=ENSBTAG00000009661|UniProtKB=Q2TBT5	RNASEH2A	Ribonuclease H2 subunit A;RNASEH2A;ortholog	endoribonuclease(PC00094)
62	BOVIN|Gene=RPA1|UniProtKB=Q0VCV0	RPA1	Replication protein A subunit;RPA1;ortholog	DNA binding protein(PC00009)
63	BOVIN|Ensembl=ENSBTAG00000021096|UniProtKB=F1N079	RTEL1	Regulator of telomere elongation helicase 1;RTEL1;ortholog	DNA helicase(PC00011)
64	BOVIN|Gene=RTEL1|UniProtKB=A4K436	RTEL1	Regulator of telomere elongation helicase 1;RTEL1;ortholog	DNA helicase(PC00011)
65	BOVIN|Ensembl=ENSBTAG00000024822|UniProtKB=A0A3Q1MN34	SETX	Senataxin;SETX;ortholog	RNA helicase(PC00032);DNA helicase(PC00011)
66	BOVIN|Gene=SMARCAD1|UniProtKB=E1B7X9	SMARCAD1	SWI/SNF-related matrix-associated actin-dependent regulator of chromatin subfamily A containing DEAD/H box 1;SMARCAD1;ortholog	DNA helicase(PC00011)
67	BOVIN|Ensembl=ENSBTAG00000017061|UniProtKB=A0A452DJD2	SMARCAD1	SWI/SNF-related matrix-associated actin-dependent regulator of chromatin subfamily A-containing DEAD/H box 1;SMARCAD1;ortholog	DNA helicase(PC00011)
68	BOVIN|Gene=SMC1A|UniProtKB=O97593	SMC1A	Structural maintenance of chromosomes protein 1A;SMC1A;ortholog	
69	BOVIN|Ensembl=ENSBTAG00000000375|UniProtKB=A6QQT5	SSRP1	FACT complex subunit SSRP1;SSRP1;ortholog	

70	BOVIN|Ensembl=ENSBTAG00000020421|UniProtKB=E1BNP8	SUPT16H	Uncharacterized protein;SUPT16H;ortholog	chromatin/chromatin-binding, or -regulatory protein(PC00077)
71	BOVIN|Gene=TIMELESS|UniProtKB=F1MQI4	TIMELESS	Uncharacterized protein;TIMELESS;ortholog	
72	BOVIN|Ensembl=ENSBTAG00000001069|UniProtKB=P67939	TP53	Cellular tumor antigen p53;TP53;ortholog	P53-like transcription factor(PC00253)
73	BOVIN|Ensembl=ENSBTAG00000006972|UniProtKB=A0A3Q1MWE7	TRIP13	Thyroid hormone receptor interactor 13;TRIP13;ortholog	chromatin/chromatin-binding, or -regulatory protein(PC00077)
74	BOVIN|Ensembl=ENSBTAG00000002224|UniProtKB=A7E320	UHRF1	E3 ubiquitin-protein ligase UHRF1;UHRF1;ortholog	ubiquitin-protein ligase(PC00234)
75	BOVIN|Ensembl=ENSBTAG00000019848|UniProtKB=Q17QB8	UNG	Uracil-DNA glycosylase;UNG;ortholog	
76	BOVIN|Gene=USP1|UniProtKB=F1MKU4	USP1	Ubiquitin carboxyl-terminal hydrolase 1;USP1;ortholog	cysteine protease(PC00081)
77	BOVIN|Gene=USP10|UniProtKB=A5PJS6	USP10	Ubiquitin carboxyl-terminal hydrolase 10;USP10;ortholog	
78	BOVIN|Ensembl=ENSBTAG00000019120|UniProtKB=E1B7F5	WDHD1	Uncharacterized protein;WDHD1;ortholog	chromatin/chromatin-binding, or -regulatory protein(PC00077)
79	BOVIN|Ensembl=ENSBTAG00000030897|UniProtKB=F6RZR1	XRCC2	Uncharacterized protein;XRCC2;ortholog	
80	BOVIN|Ensembl=ENSBTAG00000026936|UniProtKB=E1BPG1	ZGRF1	Uncharacterized protein;ZGRF1;ortholog	

Table S2. Real-time PCR primers targeting bovine MMR genes.

Gene	Primer sequence		Size of production (bp)	efficiency[a] (%)	R^{2b}
	Forward	Reverse			
MSH2	CCTCCAAGGAGAATGATTGG	AACACCAATGGAAGCAGACA	110	96.1	1.0
MSH3	CCGGAAACTGACTGCTCTTT	TCAGTCATTACCTCATCGACATTT	105	99.3	1.0
MSH6	GAGTTGGCAGTATGTGATGAGC	TCACTCTCAATGTCATTTTCCTC	105	99.4	1.0
PCNA	AGGGCTTCGACACTTACCG	R-ATGTCTTCATTGCCAGCACA	97	101.4	1.0
PMS2	AGCGTGCAGCAGCTATTTTA	R-GATGCAGTATGCCTGTAACACC	108	101.5	1.0
EXO1	GCCAGAGAATGTTTCATACGG	R-GGGGCCACAAGACAATCTAC	101	105.2	1.0
UNG	CTGTTTACCCTCCCCCTCAC	R-GGTCCGTGATATGGATCCTG	100	96.6	1.0
GAPDH	TTCAACGGCACAGTCAAGG	R-ACATACTCAGCACCAGCATCAC	119	-	1.0

[a]Efficiency and R^{2b} indicate the amplification efficiency of real-time PCR within 4 ng-0.5 ng of the template genome, and the slope of the graph obtained by plotting based on the products amplified by each primer set and the number of cycles (Efficiency) and correlation (R^2). In addition, the efficiency of each gene shows the relative amplification efficiency when the GAPDH gene as an endogenous control is set to 1.

Table S3. Real-time PCR primers targeting human MMR genes.

Symbol	Primer sequence		Size of production (bp)	efficiency[a] (%)	R^{2b}
	Forward	Reverse			
MSH2	CCAAGGAGAATGATTGGTATTTG	ACAACACCAATGGAAGCTGA	110	96.6	0.9997
MSH3	GATGCAGTTTTGTGTGTGGAA	AAAGTTGTGATCTAAATGGCAATAAA	109	97	0.9993
MSH6	AGCCCTCAGAGCCAGAAGA	GCTGTACTTCCTCTTCACTCTCAA	106	91.7	0.999
PCNA	TGAAGTTGATGGATTTAGATGTTGA	TCTCGGCATATACGTGCAAA	104	97.3	0.9993
PMS2	GCCCCAGCATAAAGGAAAAT	ACTAGGGGGCAGCTGAACA	87	97.6	0.9997
EXO1	TAGCAAAGGCATGCAAAGTC	CCTCTGGTACCGTGATATTCATC	106	98.4	0.9998
UNG	CCCACACCAAGTCTTCACCT	GAGCCCGTGAGCTTGATTAG	107	10.2	0.9987
GAPDH	AGCCACATCGCTCAGACAC	GTTAAAAGCAGCCCTGGTGA	93	-	0.9986

[a]Efficiency and R^{2b} indicate the amplification efficiency of real-time PCR within 4 ng-0.5 ng of the template genome, and the slope of the graph obtained by plotting based on the products amplified by each primer set and the number of cycles (Efficiency) and correlation (R^2). In addition, the efficiency of each gene shows the relative amplification efficiency when the GAPDH gene as an endogenous control is set to 1.

Table S4. Real-time PCR primers targeting porcine MMR genes

Symbol	Primer sequence		Size of production (bp)	efficiency[a] (%)	R^{2b}
	Forward	Reverse			
MSH2	GCTGTTGAAAGGCAAAAAGG	AAAAACTTGATTACAGCAGACAATG	102	104.8	0.9974
MSH3	ATCTTCTCCGTGCCAAGAAT	CTGTGATGATCCACAATGACC	95	98.8	0.9904
MSH6	CTGGCAGGCTGCTGTAGAGT	CTGGACGACACATAGGACCA	96	102.5	0.9995
PCNA	GTGGAGAACTCGGAAATGGA	AAGTTAGCTGAACTGGTTCATTCAT	105	104.1	0.9995
PMS2	GAGTTTGGCCTCAGCTGTTC	TGTCTGTCGGTTGAACTCCTC	105	100.4	0.9995
EXO1	CCAGAGAATGTTTTACCCGTTC	GTACGGAGCCACCAAACAGT	105	100.2	0.9992
UNG	CGCACACCAAGTCTTCACAT	GGTCCGTGATATGGATCCTG	88	100.2	0.9972
GAPDH	CTGAGACACGATGGTGAAGG	ACAATGTCCACTTTGCCAGA	97	-	0.9993

[a]Efficiency and R^{2b} indicate the amplification efficiency of real-time PCR within 4 ng-0.5 ng of the template genome, and the slope of the graph obtained by plotting based on the products amplified by each primer set and the number of cycles (Efficiency) and correlation (R^2). In addition, the efficiency of each gene shows the relative amplification efficiency when the GAPDH gene as an endogenous control is set to 1.

Table S5. Real-time PCR primers targeting bat MMR genes.

Symbol	Primer sequence		Size of production (bp)	efficiency[a] (%)	R^{2b}
	Forward	Reverse			
MSH2	GGATATTGCAGCAGTCAGAGC	CAATGCAGCCAGAGATTGAG	82	96.7	0.9981
MSH3	ACAGAGGCGCTCATCCAC	CCTGGAAAGCATGGCTGTAT	106	96.8	0.9949
MSH6	GGCTGCTGTAGAGTGCATTG	CTGGACGACACATAGGACCA	89	104.7	0.9965
PCNA	TTTTCTGCAAGTGGGGAACT	GCTGAACTGGCTCATTCATCT	109	98.8	0.9999
PMS2	GCCACAAGGAGTTTCAAAGG	CGTGGAAATGATGCAGTACG	83	98.8	0.994
EXO1[c]	–	–	-	-	-
UNG	CCATCCTGGTCATGGAGACT	TTGTGAGAATTGGCCTGATG	99	94.1	0.9948
GAPDH	TGCACCACCAACTGCTTG	GTCTTCTGGGTGGCAGTGAT	107	-	0.9999

[a]Efficiency and R^{2b} indicate the amplification efficiency of real-time PCR within 4 ng-0.5 ng of the template genome, and the slope of the graph obtained by plotting based on the products amplified by each primer set and the number of cycles (Efficiency) and correlation (R^2). In addition, the efficiency of each gene shows the relative amplification efficiency when the GAPDH gene as an endogenous control is set to 1.

[c]MMR gene expression was not measured in Tb1Lu cells due to the primer targeting EXO1 gene did not amplify.
